# Short-interval intracortical inhibition and facilitation targeting upper and lower limb muscles

**DOI:** 10.1038/s41598-021-01348-6

**Published:** 2021-11-09

**Authors:** Natalie Mrachacz-Kersting, Andrew James Thomas Stevenson, Ulf Ziemann

**Affiliations:** 1 Institute of Sport and Sport Science, Albert-Ludwigs University Freiburg, Freiburg, Germany; 2 Department of Health Science and Technology, Aalborg University, 9220 Aalborg Ø, Denmark; 3 Department of Neurology and Stroke, University of Tübingen, Tübingen, Germany; 4 Hertie Institute for Clinical Brain Research, University of Tübingen, Tübingen, Germany

**Keywords:** Motor cortex, Neural circuits

## Abstract

Transcranial magnetic stimulation (TMS) can be used to study excitability of corticospinal neurons in human motor cortex. It is currently not fully elucidated if corticospinal neurons in the hand vs. leg representation show the same or different regulation of their excitability by GABAAergic and glutamatergic interneuronal circuitry. Using a paired-pulse TMS protocol we tested short-interval intracortical inhibition (SICI) and short-interval intracortical facilitation (SICF) in 18 healthy participants. Motor evoked potentials were evoked in one hand (abductor digiti minimi) and one leg muscle (tibialis anterior), with systematic variation of the intensities of the first (S1) and second (S2) pulse between 60 and 140% resting motor threshold (RMT) in 10% steps, at two interstimulus intervals of 1.5 and 2.1 ms. For the hand and leg motor representations and for both interstimulus intervals, SICI occurred if the intensities of S1 < RMT and S2 > RMT, while SICF predominated if S1 = S2 ≤ RMT, or S1 > RMT and S2 < RMT. Findings confirm and extend previous evidence that the regulation of excitability of corticospinal neurons of the hand versus leg representation in human primary cortex through GABAAergic and glutamatergic interneuronal circuits is highly similar, and that corticospinal neurons of both representations are activated by TMS transsynaptically in largely identical ways.

## Introduction

Paired-pulse transcranial magnetic stimulation (ppTMS) at short interstimulus intervals (ISI) of 5 ms or less can be used to test short-interval intracortical inhibition (SICI)^1–3^ and short-interval intracortical facilitation (SICF)^4–6^. Pharmacological experiments indicate that SICI is a marker of gamma-amino butyric acid (GABA) type A receptor (GABAAR) mediated inhibition^7–9^. The physiology of SICF, on the other hand, is related to the neural elements responsible for the generation of I-waves^10,11^, i.e., the indirect transsynaptic excitation of corticospinal neurons in the motor cortex through excitatory interneurons^12^. SICI is typically elicited with the intensity of the first stimulus (S1) in the ppTMS protocol below resting motor threshold (RMT) and the second stimulus (S2) above RMT^2,3^. In contrast, SICF occurs if either the intensities of both stimuli are slightly below or equal to RMT^5^, or S1 is above RMT and S2 below^6^. The presence of SICF in a given ppTMS setting of S1 and S2 intensities and ISI may result in an apparent reduction of SICI^13^. SICI and SICF show clinical utility as abnormalities of both measures have been demonstrated in a broad variety of neurological disorders^14^.

Almost all SICI and SICF studies have been conducted in the hand representation of the primary motor cortex, i.e., recording motor evoked potentials (MEP) by electromyography (EMG) from a hand muscle contralateral to the stimulated motor cortex. In contrast, the literature on SICI and SICF measured in leg muscles is scarce (e.g.^15–18^). Furthermore, the expression of SICI and SICF in hand vs. leg muscles has not been compared in the same individuals with very few exceptions^19,20^. This comparison could address the question to what extent the excitability of corticospinal neurons in the hand versus leg representations of motor cortex are regulated similarly or differently.

Here we test this question by a sophisticated previously described ppTMS approach with systematic variation of S1 and S2 intensities (9 different intensities each, resulting in a matrix of 9 × 9 = 81 intensity conditions) at two different ISI (1.5 and 2.1 ms)^8^, recording from one hand muscle (abductor digiti minimi) and one leg muscle (tibialis anterior) in 18 healthy young adult subjects. The ISI of 1.5 ms was chosen since it has been shown to induce pronounced SICF when S1 > RMT and S2 < RMT^6^, while 2.1 ms is known to produce distinct SICI when S1 < RMT and S2 > RMT^21^.

## Materials and methods

Eighteen healthy participants (4 females, mean age: 23.3 ± 2.1 years; all right side dominant) without any known physical or neurological disorders attended four experimental sessions separated by at least one week. The study was approved by the Ethics Committee of Northern Jutland (N-20150060) and was performed according to the declaration of Helsinki. All participants provided written informed consent prior to participation. The procedure for each session was the same, the only difference being the target muscle (abductor digiti minimi (ADM) or tibialis anterior (TA)), and the interval between the pairs of TMS stimuli (1.5 or 2.1 ms). Two participants took part in all four sessions, thus there were a total of 10 participants for each condition. This number was based on an effect size calculation^22^. An analysis of previous data has shown that the MEP amplitude has on average, a standard deviation of approximately 0.35 mV (this is considered for different stimulation intensities). The MEP amplitude is the primary measure in the study and the power was set to 0.80 with a significance level of p ≤ 0.05. The recommended number of participants for each session was found to be 8. The variance will increase if the subject group responses are more heterogeneous than those from the pilot study. To compensate for this possibility, we used 10 participants per experiment.

Bipolar surface electrodes (Ambu Neuroline 720, Ambu A/S, Denmark) were placed on the ADM or TA of the right dominant side according to SENIAM guidelines (seniam.org). The electromyography (EMG) amplifier pod (Bandwidth 16–550 Hz) supplied by Rogue Research Inc. as part of the Brainsight™ system (Rogue Research inc.) was used to record EMG data at a sampling rate of 3 kHz. TMS was applied over the left hemisphere using a Magstim BiStim^2^ (Magstim Company, Dyfed, UK) with a monophasic current waveform, connected to a figure-of-eight coil (custom-made Magstim Alpha Coil Flat Range (Coated), diameter of each wing: 70 mm).

The optimal placement of the TMS coil was determined using 50% of the maximum stimulator output (MSO) and 75% MSO for the ADM and TA muscles, respectively. These intensities had to be increased in some individuals in order to exceed the motor threshold. For ADM, the starting position was approximately 5.5 cm lateral to the vertex and 0.5 cm anterior to the interaural line with the coil handle pointing backward and rotated 45° away from midline, i.e., the induced current in motor cortex was oriented from lateral-posterior to medial-anterior, an orientation that is optimal for indirect activation of corticospinal neurons^23^. For TA, the coil was initially placed over the vertex such that the induced current direction in the targeted left hemisphere was from medial to lateral, i.e., the optimal orientation for eliciting MEPs^24^ and SICI^15^ in the leg representation. Three consecutive stimuli were applied and the peak-to-peak amplitude of the MEP in the target muscle monitored online. This was repeated for 3–5 positions and the site that resulted in the largest and most consistent MEPs was taken as the hotspot. The coil position was maintained by marking this spot using Brainsight™. Next, RMT was established, which was the lowest stimulation intensity that produced MEPs with a peak-to-peak amplitude of at least 50 μV while the target muscle for the respective session was at rest, in 5 out of 10 consecutive trials^25^.

Pairs of transcranial magnetic stimuli with an interstimulus interval (ISI) of either 1.5 or 2.1 ms were applied. The intensities of the first (S1) and second (S2) stimulus in all trials were varied randomly in steps of 10% RMT between 60–140% RMT. This way, the paired-pulse conditions consisted of all possible combinations of S1 and S2 intensities (i.e., 81 conditions)^8^. In addition, nine single-pulse conditions were tested at the nine different intensities (i.e., 9 conditions). Five trials were performed for each paired-pulse TMS condition and ten trials for each single stimulus TMS condition. The different conditions were applied in pseudo-randomized order. The inter-trial interval varied between 7–10 s.

The MEP peak-to-peak amplitudes were extracted for each trial and subsequently averaged for each stimulus pair condition. The interaction between S1 and S2 was expressed as the ratio of the MEP amplitude elicited by paired-pulse TMS (MEP_S1+S2_) over the algebraic sum of the MEP amplitudes produced by the corresponding single stimuli (MEP_S1_ + MEP_S2_)^8^.

Paired *t*-tests (two-tailed) were performed for each muscle and ISI comparing the observed paired-pulse MEP amplitudes (MEP_S1+S2_) and the algebraic sum of the single-pulse MEP amplitudes (MEP_S1_ + MEP_S2_) in order to identify stimulus conditions resulting in significant SICI or SICF. Multiple comparisons were corrected using the Holm-Bonferroni method^26^.

The similarity of the inhibitory and facilitatory interactions in all 81 cells of the matrix defined by the 9 × 9 stimulus intensity conditions of S1 and S2 was compared between ADM and TA at the ISI of 1.5 ms and 2.1 ms by using linear regression statistics. Correlation coefficients were calculated, and the null hypothesis of independent regulation of inhibitory and facilitatory interactions between the two muscle representations was rejected if *p* < 0.05.

## Results

RMT for the ADM muscle was 51 ± 10% MSO and 42 ± 4% MSO for the 1.5 and 2.1 ms ISIs, respectively, while the RMT for the TA muscle was 68 ± 9% MSO and 64 ± 7% MSO for the 1.5 and 2.1 ms ISIs, respectively. The group differences for a given muscle between the two ISIs are explained by the largely non-overlapping groups of participants (see “Materials and methods”).

**Figure 1:**
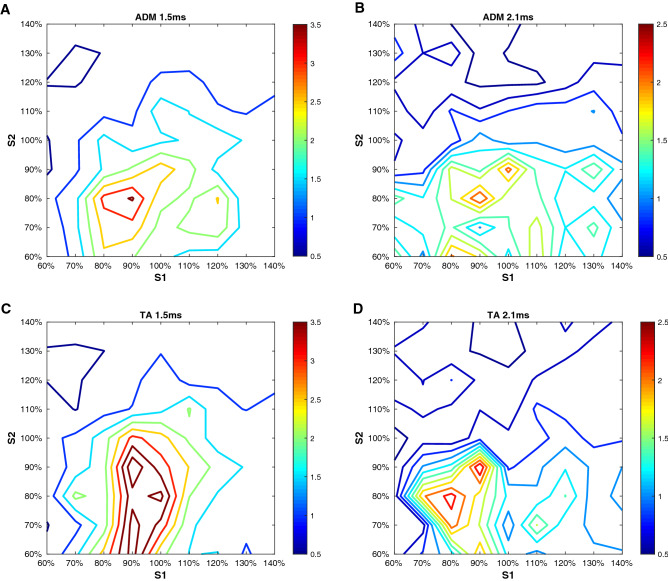
Short-interval intracortical inhibition and facilitation as a function of stimulus intensity and interstimulus interval in the resting abductor digiti minimi (ADM) and tibialis anterior (TA). (**A**-**B**) refer to the ADM at an interstimulus intervals (ISI) of 1.5 (**A**) and 2.1 ms (**B**). (**C-D)** contain the data for the TA at an ISI of 1.5 (**C**) and 2.1 ms (**D**). In each plot, the stimulus intensity of the first stimulus (S1, x-axis) and the second stimulus (S2, y-axis) is related to the resting motor threshold (RMT) of the ADM (**A-B**) or the tibialis anterior (**C-D**). For each condition, the interaction between S1 and S2 is expressed as the percentage of motor evoked potential (MEP) amplitudes produced by paired TMS (MEP_S1+S2_) over the arithmetic sum of the MEP produced by the single stimuli (MEP_S1_ + MEP_S2_). Inhibitory (< 1.0) and facilitatory interactions (> 1.0) are color-coded (see color bars). Each plot shows the average data across 10 participants. The figure was created using Matlab (Mathworks, version 2020).

When expressing the interaction between S1 and S2 as MEP_S1+S2_/(MEP_S1_ + MEP_S2_), the activation of inhibitory and excitatory circuits was found, for both muscles in a similar way, to be dependent on the intensities of S1 and S2, and the ISIs (Fig. 1).

For both muscles and ISIs, when S1 < RMT and S2 > RMT, an inhibitory interaction of S1 and S2 was seen. For the typical setting for S1 and S2 used in upper limb muscles, S1 = 80% RMT and S2 = 120% RMT, SICI was seen in 9/10 participants for the ADM at both ISIs, while SICI was seen in 8/10 and 6/10 participants for the TA at the 1.5 ms and 2.1 ms ISIs, respectively.

For the ADM at the 1.5 ms ISI, the optimal stimulation condition inducing SICI in all ten participants was S1 = 80% RMT and S2 = 140% RMT, which suppressed the MEPs to 52 ± 26% (*p* = 0.001, not significant when corrected for multiple comparisons). For the ADM at the 2.1 ms ISI, the optimal stimulation condition inducing SICI in all participants was S1 = 80% RMT and S2 = 130% RMT, which suppressed the MEPs to 39 ± 16% (*p* = 0.009, not significant when corrected for multiple comparisons).

For the TA at the 1.5 ms ISI, the optimal stimulation condition inducing SICI in all ten participants was S1 = 70% RMT and S2 = 130% RMT, which suppressed the MEPs to 49 ± 20% (*p* < 0.0006). For the TA at the 2.1 ms ISI, the optimal stimulation condition inducing SICI in all participants was S1 = 80% RMT and S2 = 130% RMT, which suppressed the MEPs to 37 ± 23% (*p* = 0.005, not significant when corrected for multiple comparisons).

When S1 was close to or equal to RMT and S2 was slightly below RMT, an excitatory interaction (i.e., SICF) of S1 and S2 occurred for both the ADM and TA muscles at the 1.5 ms ISI. The typical settings in upper limb muscles, S1 = 120% RMT and S2 = 90% RMT and S1 = S2 = 100% RMT, resulted in SICF in 8/10 participants each for the ADM and 9/10 and 7/10 participants, respectively, for the TA. However, for the 2.1 ms ISI for the ADM and TA, these combinations of S1 and S2 resulted in SICF in only 1–5/10 participants.

For the ADM at the 1.5 ms ISI, the optimal stimulation condition inducing SICF in all ten participants was S1 = 100% RMT and S2 = 90% RMT, which facilitated the MEPs to 349 ± 374% (*p* = 0.016, not significant when corrected for multiple comparisons). For the TA at the 1.5 ms ISI, the optimal stimulation condition inducing SICF in all ten participants was S1 = 90% RMT and S2 = 90% RMT, which facilitated the MEPs to 448 ± 601% (*p* = 0.005, not significant when corrected for multiple comparisons).

For the ADM and TA at the 2.1 ms ISI, none of the combinations of S1 and S2 induced a significant facilitation effect even before the Holm-Bonferroni correction was applied (all *p* > 0.07).

**Figure 2:**
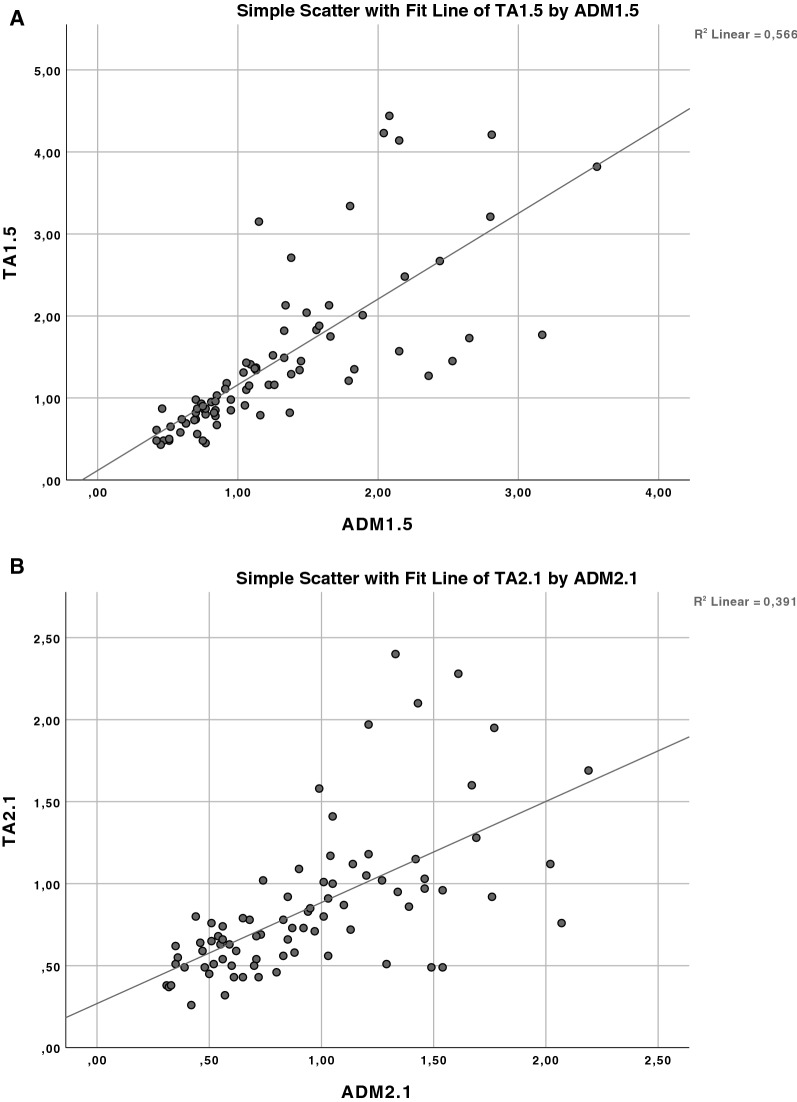
Similarity of short-interval intracortical inhibition and facilitation between the abductor digiti minimi (ADM) and tibialis anterior (TA) representations. The similarity of the inhibitory and facilitatory interactions in all 81 cells of the matrix defined by the 9 × 9 intensity conditions of the first and second stimulus in the paired-pulse TMS protocol was compared for the ADM and TA at the interstimulus intervals of 1.5 ms (**A**) and 2.1 ms (**B**) by linear regression statistics. In the regression plots, values < 1.0 indicate short-interval intracortical inhibition, and values > 1.0 short-interval intracortical facilitation. Note that the high correlation coefficients of r = 0.752 (**A**) and r = 0.626 (**B**) provide strong evidence that the null hypothesis that the inhibitory and facilitatory interactions for the two muscle representations are regulated independently can be rejected (both *p* < 0.0001).

The similarity analysis of the inhibitory and facilitatory interactions between the ADM and TA representations in all 81 cells of the matrix defined by the 9 × 9 stimulus intensity conditions of S1 and S2 using linear regression statistics revealed correlation coefficients of r = 0.752 for the ISI of 1.5 ms, and r = 0.626 for the ISI of 2.1 ms, providing strong evidence that the null hypothesis that the inhibitory and facilitatory interactions of the two muscle representations are regulated independently can be rejected with high confidence (both *p* < 0.0001, Fig. 2).

## Discussion

For the hand representation in motor cortex, we have replicated the findings of Ilic and colleagues (cf. Figure 1 in^8^): SICI occurred if the intensities of S1 < RMT and S2 > RMT, and SICF predominated if S1 = S2 ≤ RMT, or S1 > RMT and S2 < RMT (Fig. 1). The novel finding is that the “territories” in the S1 x S2 intensity matrix where SICI and SICF occurred in the leg representation were highly similar to those of the hand representation (Figs. 1, 2), for both ISI of 1.5 and 2.1 ms. This strongly suggests that interneuron-mediated synaptic control of excitability of corticospinal neurons in hand and leg representations of the primary motor cortex is similar. SICI is an established marker of GABAAR receptor mediated synaptic inhibition. The evidence was driven mainly by pharmaco-TMS studies that demonstrated a significant increase of SICI by positive allosteric modulators of the GABAAR, such as benzodiazepines (for review^27^). In contrast, SICF is a marker of excitability of chains of excitatory interneurons^8,28^. Excitation of these interneurons through the TMS pulse leads to glutamatergic transsynaptic activation of corticospinal neurons^29^.

The present findings with strong similarity of SICI and SICF between hand and leg representations of primary motor cortex are mirrored by similarities in GABAAR and glutamate receptor densities throughout the latero-medial extent of the primary motor cortex containing the hand and leg representations, as determined by autoradiography in monkeys^30,31^.

Moreover, single motor unit recordings have provided evidence that the corticospinal projections to the alpha motoneurons of hand muscles and the TA muscle are largely monosynaptic, with similar sizes of excitatory postsynaptic potentials elicited by TMS of the hand and leg representations of motor cortex^32,33^. The similar organization of the motor cortex output systems to hand muscles and the TA muscle provides a reasonable basis to assume that the synaptic excitability of the corticospinal neurons is also regulated by largely identical networks of inhibitory and excitatory interneurons. Accordingly, SICI and SICF were demonstrated in a few studies for the leg representation before (e.g.^15–18^)but these findings were not directly compared to measurements in the hand representation with a few exceptions^19,20^. However, the study of Chen et al.^20^ did not include the TA muscle but other leg muscles with probably less direct corticospinal projections, and SICF was not tested. The study by Chen and Garg^19^ demonstrated with a ppTMS protocol that three I-wave facilitation (SICF) peaks occurred at discrete interstimulus intervals that were identical between a hand muscle and the TA muscle (see below) but did not systematically test variations of S1 and S2 intensities, which is the focus of the present study.

The findings of the present study are important as they demonstrate a similar, predominantly transsynaptic activation of corticospinal neurons through I-waves in both representations and a similar regulation of their excitability by GABAAergic interneurons. This is at variance with early investigations that provided evidence for a predominantly direct, i.e., non-synaptic activation of corticospinal neurons of the leg representation^34^. However, this view was rectified by other studies that demonstrated with epidural mid-thoracic spinal cord recordings predominantly the elicitation of the I1-wave with TMS at threshold intensity, and sometimes a D-wave and even an I2-wave, and at higher stimulation intensities recruitment of more late I-waves, verifying that TMS activates corticospinal neurons of the leg representation predominantly indirectly^35^. These findings were confirmed by single motor unit recordings from the TA muscle, again showing predominant elicitation of the I1-wave with close to motor threshold intensity and then late I-waves with higher stimulus intensities, while a D-wave was rarely recruited^24^. Moreover, ppTMS experiments demonstrated very similar SICF for a hand muscle and the TA with variation of the interstimulus interval: facilitation occurred in three distinct peaks at ISIs of 0.9–1.7, 2. 5–3.5, and 4.1–5.1 ms^19^. This study also demonstrated similar amounts of SICI for the hand and leg representations^19^. Moreover, epidural spinal recordings demonstrated that SICI of the leg representation resulted in inhibition specifically of late I-waves^36^, very similar to the observations when stimulating the hand representation^37^.

In conclusion, this study confirms and extends the evidence that the regulation of excitability of corticospinal neurons of the hand versus leg representation in human primary cortex through GABAAergic and glutamatergic interneuronal circuits is highly similar, and that corticospinal neurons of both representations are activated by TMS in largely identical ways.

## Data Availability

All data used for this study are available from the authors upon request.
